# Supplementary material for: Are carbon emissions trading and green financial instruments synergistic? -Comprehensive quantitative research based on content analysis
Source: PLoS One. 2024 Mar 7;19(3):e0298601. doi: 10.1371/journal.pone.0298601 (PMC10919601; doi:10.1371/journal.pone.0298601)
Supplement: S1 Data — (ZIP) [file pone.0298601.s001.zip › Data/地级市Z_F工作报告.zip!/╔╜╢1⁄2/║╩╘≤╩╨/└┤╫╘╣1⁄2╓┌║┼í╛┬φ┐╦╩2╛▌═°í┐_2006.docx]

各位代表:

　　现在,我代表市人民政府向大会作政府工作报告,请予审议,并请市政协委员和其他列席会议的同志提出意见。

　　一、“十五”时期的工作回顾

　　“十五”时期是我市历史进程中极不平凡的时期。五年来,在省委、省政府和市委的正确领导下,各级政府团结依靠全市人民,高举邓小平理论和“三个代表”重要思想伟大旗帜,认真贯彻科学发展观,努力克服洪涝灾害和非典、禽流感等疫情的影响,全力推进经济社会快速健康发展,圆满完成了“十五”计划确定的各项任务目标。

　　————经济发展速度明显加快。2005年实现生产总值451亿元,“十五”年均增长13.1%,比“九五”年均加快6.5个百分点。地方财政收入达到21.2亿元,按可比口径年均增长21.6%,比“九五”年均加快6.2个百分点。

　　————产业结构进一步优化。三次产业比调整为34:43:23,二三产业比重提高16个百分点,其中工业增加值比重提高12.9个百分点,产业占比实现了由一、二、三向二、一、三的历史性转变。

　　————发展后劲显著增强。累计完成固定资产投资1011亿元,是“九五”的近4倍,年均增长44.2%。对全市发展具有重大影响的巨野煤田开发项目正式启动,一大批新建和扩建重点项目相继投产。园区建设实现了重大突破。

　　————城乡面貌发生较大变化。市县两级城市建设累计投资80亿元,其中市区累计完成财政性投入18亿元。全市城市规划、建设、经营、管理水平大幅度提高,城市功能显著增强,农村“路水电医学”条件有了较大改善。

　　————人民生活水平稳步提高。城镇居民人均可支配收入和农民人均纯收入分别达到7342元和3092元,比2000年增加2440元和1025元。城乡居民储蓄余额314.4亿元,年均增长12.6%。就业再就业规模不断扩大,社会保障水平进一步提高。

　　————社会事业全面发展。科技、教育、文化、卫生、体育、广播电视等社会事业有了长足发展,人口、资源和环境工作不断加强,社会管理和公共服务水平有新的提高,精神文明创建活动深入开展,全市上下的思想观念和精神面貌发生了深刻变化,干事创业的氛围更加浓厚。

　　过去的五年,是我们应对挑战、抢抓机遇、乘势而上的五年,是凝聚力量、扎实苦干、在突破菏泽征程上迈出较大步伐的五年。今天的菏泽,经济快速发展,社会和谐稳定,人民安居乐业,已经站在一个新的历史起点上!

　　在过去的五年中,我们主要做了以下几个方面的工作:

　　(一)抢抓历史机遇,推动经济快速健康发展。充分利用省委、省政府实施“突破菏泽”战略的机遇、国家加强宏观调控的机遇和国内外产业转移的机遇,坚持以扩大投资为总抓手,全力加快经济发展。一是大力开展招商引资。发挥我市土地、水、电、劳动力资源丰富的综合优势,在组织搞好产业和项目策划的基础上,利用牡丹花会、林交会两个招商平台,面向日韩、港台和东南沿海地区,开展了大规模的招商活动,收到了较好效果。二是努力争取上级投资。完成了日照至东明高速公路(菏泽段)工程、骨干河道治理工程、城乡电网改造工程,开工建设了菏泽至兰考、济南至菏泽高速公路(菏泽段)等一批重大基础设施工程,在农业、教育、卫生等方面实施了一批国债资金支持项目。三是积极推进东企西进。共签约东西联动项目141个,完成投资54亿元。四是加快园区建设。拉开了菏泽开发区建设大框架,各县工业园区在东部八市对口帮扶下加快了建设步伐。

　　(二)坚持工业立市,着力调整优化经济结构。根据市委关于加快工业化进程的决定,大力培植优势产业和骨干企业,加快发展区域板块经济。全市规模以上工业企业达到821家,新增572家,完成增加值117.8亿元,年均增长30%。利税过千万元的企业达到36家,比2000年增加14家。菏泽电厂、东明石化等30家重点企业较好地发挥了对财政经济的支撑作用。加强经济运行调度,及时解决经济运行中的困难和问题。在努力加强企业信用建设的同时,积极促进银企合作,连续举办了四次银企合作项目洽谈会,共签约项目398个,协议贷款金额423.6亿元。注重加强企业创新能力建设,鼓励支持企业技术改造和新产品开发。全市建立省级企业技术开发中心8个,培育省级以上名牌产品25个。

　　(三)以增加农民收入为核心,加大“三农”工作力度。认真贯彻落实胡锦涛总书记视察菏泽时的重要讲话精神,切实把解决“三农”问题作为全部工作的重中之重。一是不折不扣地落实各项支农惠农政策,有效地调动了广大农民发展农业生产的积极性,促进了粮食生产能力的提高。2005年全市粮食总产达到410.1万吨,比2000年增长16.2%,创1997年以来的最高水平。二是坚定不移地推进农业结构调整。积极发展优质高效经济作物,加快林业、畜牧业发展步伐。大力扶持龙头企业,促进农业产业化经营。2005年林牧业在农业增加值中的比重达到23.3%,比2000年提高4.7个百分点;规模以上农业龙头企业达到460家,五年增加300家。三是千方百计扩大劳务输出。借助“西输东接”机遇,加快人力资源市场建设,组织开展劳务推介活动,强化职业技能培训,劳务输出规模和层次有了新的提高。全市劳务输出人员达到126万人,比2000年增加70万人。四是多方筹资加大对农业农村基础设施的投入。在对骨干河道进行大规模治理的基础上,广泛开展了以“三沟”治理为重点的农田水利建设;完成了市管河道一二类危桥改造;新建改造农村公路12049公里,基本实现了村村通油路和村村通客车,改善了农村生产生活条件。

　　(四)坚持“四高”原则,努力提高城市化水平。以建设区域中心城市为目标,高起点编制了新一轮城市总体规划,完成了10多项标志性地段详细规划。加大了对城市道路、防汛、排污、供水、供气、供热、电力、电信等基础设施的投入,基本完成了环城公园、赵王河公园、污水处理厂、中国林展馆等一批重点工程,带动了房地产业和商业服务业发展。实施了城市“绿、亮、清”工程,推行环卫等城市管理体制改革,取得了明显成效。同时,加大了各县县城和中心镇建设力度。通过经营城市,实施节点开发,带动土地增值,初步形成了城市资本投入、增值、再投入、再增值的良性发展机制。

　　(五)不断深化改革,努力扩大对外开放。扎实推进农村税费改革,全市农民累计受益5.3亿元,人均72元。进一步深化了企业改革,强化了国有资产监管。在实行收支两条线和会计委派制的基础上,实施了六项财政综合改革,依法治税理财水平明显提高。坚持市场化取向,加大了投资体制和市政公用事业改革力度。放宽了投资领域,激发了民营经济的活力。全市个体私营经济生产总值和税收已分别占到全市生产总值和税收收入的39%和34%。积极适应我国入世、国家出口退税体制改革,全方位扩大对外开放,五年实际利用外资4.5亿美元,比“九五”增长2.4倍;2005年进出口总额5.2亿美元,比2000年增长6.7倍。

　　(六)重视解决关系群众切身利益的实际问题,大力发展社会事业。高度重视就业再就业工作,累计新增城镇就业33万人。努力推进养老保险扩面征缴,争取省里的资金支持,确保了企业离退休人员养老金和失业人员保险金按时足额发放。在全省率先实行了“两证一卡”制度,保障了农村困难群众的基本生活需要。城镇低保标准提高到140元,实现了动态管理下的应保尽保。认真解决拖欠企业职工和农民工工资问题。去年启动的以住房、就业、卫生等为内容的十大民生工程,正在积极推进并取得初步成效。以县为主的农村义务教育管理体制初步形成,投资3.9亿元改造农村中小学危房107.8万平方米,职业技术教育快速发展,组建了菏泽学院,结束了我市没有本科院校的历史。科技创新能力不断提高,“十五”期间取得重要科技成果421项,专利771项。积极引入市场机制,加快文化、体育、广播电视、新闻出版的产业化步伐。基本建成了市县乡村四级疾病预防控制和卫生监督网络。东明县新型农村合作医疗试点工作进展顺利,农民参合率达到91%。人口和计划生育工作成效显著,年均人口自然增长率6.86‰。妇女儿童、老年人、残疾人权益得到较好保障,民族、宗教、人防、史志、档案、地震、气象等工作都取得了新的成绩。

　　(七)正确处理改革发展稳定关系,积极营造良好的社会环境。

　　注重把改革的力度、发展的速度和社会可承受程度统一起来,在确保社会稳定的前提下深化改革、推动发展。在涉及群众切身利益的问题上,特别是城镇拆迁、农村征地和破产企业重组等方面,严格执行国家政策,维护群众的合法权益。严厉打击制售假冒伪劣商品等经济犯罪活动。坚持工作重心下移,及时化解社会矛盾。建立健全群专结合、群防群治的治安防范体系,深入开展打霸治痞、打击盗窃牲畜和暴力犯罪等“三大战役”,大力推进平安菏泽建设。认真落实安全生产责任制,加强对重点行业、重点领域的监管,安全生产形势明显好转。

　　(八)坚持依法行政,切实加强政府自身建设。认真贯彻实施《行政许可法》和《全面推进依法行政实施纲要》,切实加强法治政府建设。加快政府职能转变,推行行政审批制度改革,建立了行政审批一站式服务体系。在各级政府和公共服务单位普遍推行了政务公开,并先后建立了市长公开电话、政府网站、政策法规咨询中心,畅通了与群众联系的渠道。按照建立“三个体系”的要求,建立健全科学民主决策机制,加强跟踪调度,加大了推动工作措施落实的力度。自觉接受人大、政协的监督,加强行政复议工作和监察审计等专门监督机关的工作。严格落实党风廉政建设责任制,加强领导干部廉洁自律,深入开展纠风工作,严肃查处行政不作为、乱作为问题。全市各级政府依法行政的水平有了明显提高。

　　各位代表!回顾“十五”期间的工作,我们深感成绩来之不易。菏泽的每一点成绩,每一点变化,无不得益于上级领导的关怀和帮助,无不凝结着全市人民的心血和汗水,无不体现着社会各界的支持与合作。在此,我代表市人民政府,向在各自岗位上辛勤劳动、做出贡献的全市人民,向所有关心和支持菏泽发展的同志们、朋友们,表示衷心的感谢并致以崇高的敬意!同时,我们也清醒地看到,我市与先进市的差距仍然较大,前进道路上还有不少困难和问题。一是经济结构不合理。规模以上工业增加值只占生产总值的26.1%,低于全省23.3个百分点,尤其是缺少具有市场竞争力的龙头企业和优势产业。二是经济增长方式粗放。企业自主创新能力不强,自主知识产权、自主创新品牌和高附加值的产品比重低,尤其是人才匮乏,劳动者素质不能适应发展的要求,投资效益有待进一步提高。三是“三农”问题仍很突出。农业基础薄弱,综合生产能力较低,农村整体发展滞后,农民增收的长效机制还没有真正建立起来。四是影响持续发展的体制性机制性障碍尚未完全消除,公平竞争的市场环境尚未真正形成,用市场手段配置生产要素的效率不高。五是财源基础薄弱,财政持续增长乏力。政府公共投入不足,社会事业发展滞后。同时,一些政府部门的工作作风、服务效率等方面还存在不少问题。对此,我们一定高度重视,认真加以解决。

　　二、“十一五”时期的目标任务

　　“十一五”是我市加快发展、实现突破的关键时期,也是全面落实科学发展观、推动经济社会发展转入科学发展轨道的关键时期。根据上级的要求和市委的意见,市政府编制了《菏泽市国民经济和社会发展第十一个五年规划纲要(草案)》。《纲要(草案)》及《说明》已印发大会,请一并予以审议。

　　综合分析各种有利条件和不利因素,《纲要》提出“十一五”时期全市经济社会发展总的指导思想是:坚持以邓小平理论和“三个代表”重要思想为指导,认真贯彻党的十六届五中全会和胡锦涛总书记、温家宝总理视察菏泽时的指示精神,坚持以科学发展观统领经济社会发展全局,按照省委、省政府实施“突破菏泽”战略的目标要求,以改革开放和科技进步为动力,以调整经济结构和转变增长方式为主线,以提高人民生活水平为根本出发点,加快工业化、城市化、农业现代化进程,促进经济又快又好发展,努力构建社会主义和谐社会。主要发展目标是:

　　————综合经济实力再上新台阶。全市生产总值达到795亿元,年均增长12%;人均达到1000美元以上。地方财政收入达到43亿元,年均增长15%。

　　————产业结构调整取得重大进展。三次产业结构调整到24:51:25,二三产业比重提高10个百分点;初步建成“三大基地”。

　　————经济增长方式明显转变。万元生产总值能源消耗年均降低3.5%,工业用水重复利用率提高到65%。高新技术产业产值占比达到25%。

　　————发展后劲进一步增强。固定资产投资年均增长25%,五年累计利用外资10亿美元。社会消费品零售额年均增长15%,进出口贸易年均增长25%。

　　————城市功能大幅提升。完成财政资金投入25亿元,市区人口达到50万人以上,城市化水平达到35%以上,初步形成具有较强辐射带动能力的区域性中心城市。

　　————居民生活水平普遍提高。城镇居民人均可支配收入达到11300元,农民人均纯收入达到4550元,年均分别增长9%和8%左右。就业规模不断扩大,社会保障体系进一步完善,社会事业全面进步,生态环境达到全省先进水平。

　　工作中重点把握好以下几个方面:

　　一是更加注重科学发展,努力实现又快又好发展。坚持把发展作为第一要务,以经济建设为中心,聚精会神搞建设,一心一意谋发展。把科学发展观贯穿到经济社会发展的全过程,转变发展理念,创新发展模式,提高发展质量,努力保持较快的发展速度、较好的发展效益和较强的发展后劲。

　　二是更加注重优化经济结构,大力推进工业化进程。工业化是不可逾越的发展阶段,是改变我市欠发达局面的治本之策,是实现突破菏泽目标的根本出路。紧紧抓住结构调整这条主线,发挥自身优势,围绕建设“三大基地”,培植“六大产业”,集中力量发展一批能够立县立市的骨干企业和名牌产品,提高我市经济的整体素质和市场竞争力。

　　三是更加注重投资质量,充分发挥投资效益。“十一五”时期是我市加速推进工业化的重要阶段,投资仍然是拉动经济增长的主要力量。要坚持不懈地把招商引资、扩大投资作为经济工作的总抓手,在努力扩大投资总量的同时,高度重视投资的结构、质量和效益,着力提高投资水平,靠扩大优质增量促进经济又快又好发展。

　　四是更加注重城乡统筹,促进协调发展。以区域中心城市建设为重点,大力推进城市化进程。围绕社会主义新农村建设,统筹城乡产业发展,增加农民收入;统筹城乡就业,促进城镇就业再就业和农村劳动力转移;统筹城乡基础设施建设,改善农村发展条件;统筹城乡社会发展,加快发展农村社会事业;统筹城乡规划建设,改善村容村貌;统筹推进城乡改革,逐步形成有利于以城带乡、城乡互动、共同发展的体制和机制。

　　五是更加注重提高自主创新能力,深入实施“科教兴菏”和“人才强市”战略。在搞好技术引进和消化吸收的同时,把提高自主创新能力作为转变经济增长方式、调整经济结构的中心环节,建立健全以企业为主体、市场为导向、产学研相结合的技术创新体系,进一步完善鼓励科技进步和自主创新的各项政策,积极探索符合菏泽特点的创新之路。全面落实教育优先发展的方针,巩固提高义务教育,大力发展职业教育,提高高等教育质量。切实加强人才队伍建设,变人口大市为人力资源强市,形成人尽其才、才尽其用的良好环境。

　　六是更加注重转变经济增长方式,建设资源节约型、环境友好型社会。把转变经济增长方式作为经济工作的一项战略重点来抓,使经济增长建立在高效利用资源、减少环境污染、保护生态环境的基础之上。坚持开发节约并重、节约优先的原则,大力推行清洁生产,发展循环经济,逐步形成节约型的生产方式和消费方式。加大污染治理力度,严格控制新的污染源,积极推进生态市建设。

　　七是更加注重经济社会协调发展,推进和谐社会建设。在加快经济发展的同时,更好地履行政府公共管理和社会服务职能,加大公共投入,完善公共服务设施,加快文化、卫生、体育等各项社会事业的发展,不断满足人民群众日益增长的物质文化需要。加强民主法制建设,完善社会管理机制,正确处理人民内部矛盾和其他社会矛盾,大力推进平安菏泽、文明菏泽和诚信菏泽建设,积极构建社会主义和谐社会。

　　八是更加注重改革开放,增强经济社会发展活力。改革开放是经济社会发展的强大动力,也是解决影响未来发展诸多矛盾和问题的必然选择。要坚持社会主义市场经济改革的方向,以体制创新和制度建设为重点,全面推进政府、农村、企业、财政、金融等领域的改革。积极实施互利共赢的开放战略,加快转变对外贸易增长方式,更加积极地利用外资,在更大范围、更广领域和更高层次上参与国际竞争与合作,更好地利用两个市场、两种资源,全面提高对外开放水平。

　　三、2006年的工作重点

　　2006年是实施“十一五”规划的开局之年,做好今年的工作,具有极为重要的意义。全市经济社会发展的主要预期目标是:生产总值增长13%;地方财政收入增长16%;全社会固定资产投资增长25%;社会消费品零售总额增长15%;外贸进出口增长25%,实际利用外资增长50%;城镇居民人均可支配收入和农民人均纯收入分别增长9%和8%;人口自然增长率控制在6.8‰以内;万元生产总值能耗降低3%。围绕实现上述目标,重点抓好以下九个方面的工作:

　　(一)进一步优化投资结构,在提高投资水平上实现新突破

　　继续把招商引资作为扩大投资的重点来抓,牢牢抓住发达国家和东南沿海地区产业转移机遇,围绕资源开发、企业重组和产业配套,有针对性地搞好项目对接,重视发挥企业的主体作用,进一步改进招商方式,增强招商实效。加强企业信用建设,完善信用担保体系,改善金融生态环境,扎实推进银企合作,力争信贷投资实现快速增长。用足用好突破菏泽政策机遇,积极争取对口帮扶单位的支持,努力推动东企西进。切实做好基础设施和社会公益事业等重点项目的前期工作,通过有选择地提供地方配套资金或财政担保等,最大限度地争取上级资金和国外贷款。继续做好已纳入国家和省“十一五”规划重点项目的前期工作,争取尽早开工建设。进一步放宽市场准入,优化服务环境,激活民间投资。支持达驰、圣奥、步长等优势企业上市融资。

　　在拓宽投资渠道、扩大投资总量的同时,着力优化资金投向,提高资本聚集度。精心策划论证一批高水平项目,引导资金投向支柱产业和骨干企业,投向农业和服务业,投向基础设施和社会事业,努力提高资本聚集度,最大限度地发挥投资效益,形成产业集群效应。完善考核办法,引导提高投资水平。严把项目审核关,严格落实投资强度、科技含量、环保标准和对财政贡献的有关规定,坚决禁止高耗能、高污染项目投资,防止盲目投资和低水平重复建设。

　　坚持工业园区化、园区城镇一体化,把园区作为扩大投资的主要载体,举全市之力建设菏泽经济开发区,借助东部八市对口帮建工业园区的机遇,加快各县工业园建设步伐。在搞好园区规划,完善园区配套设施,改善项目落地条件的基础上,加大招商引资力度,推动项目向园区集聚,努力把园区建设成为全市的产业基地、创新基地、财源基地和就业基地。力争年内园区完成投资275亿元,落地项目650个,实现投产达效企业350个。进一步创新园区管理体制,完善园区服务体系,着力营造亲商、安商、富商的良好环境。

　　(二)着力培育“三个一批”,千方百计加快工业发展

　　面对日趋激烈的市场竞争,适应经济全球化、产业化、集约化进程不断加快的新形势,要站在战略全局的高度,着力培育具有比较优势和地方特色的支柱产业、骨干企业和名牌产品,加快工业经济的发展,增强全市经济的整体实力和市场竞争力。重点加快巨野煤田六对矿井及大型电源项目、煤化工项目建设,尽快发挥其在全市经济发展中的支撑带动作用;做好东明石化大炼油项目的前期工作,促进洪业化工、玉皇实业等石化企业快速膨胀;推动睿鹰、圣奥、单县有机化工等企业加快新产品研发,提升在全国同行业中的龙头地位。充分发挥晨鸣、森利、曹普、兴安等骨干企业的带动作用,重点建设一批精深加工项目,带动林业化工、机械和包装产业加快发展。以华瑞、鲁花、银香伟业、青啤、富仕达等企业为重点,抓好精深加工,提高农副产品加工档次和附加值。大力培植艺达、百隆、银河、华灵等龙头企业,拉长产业链条,搞好产业配套,带动纺织产业升级。依托达驰、华星等企业,加强与国内外大企业的合资合作,进一步提高机械工业产品的科技含量,扩大产业规模。力争年内销售收入过亿元的企业超过100家;争创一批省级、国家级名牌产品。

　　积极利用高新技术和先进适用技术改造传统产业,加快淘汰落后的生产技术和工艺,提升支柱产业、骨干企业和重点产品的技术水平。力争年内开发20项关键技术、40个创新项目和600个新产品。加快生物制药、煤化工、油气化工和节能环保等高新技术产业化步伐,力争高新技术企业达到60家,规模以上高新技术企业产值占比达到16%以上。以市开发区西区为依托,加快筹建省级高新技术产业区。

　　依托产业基础和资源优势,积极培育特色产业群体,加快发展集特色产业、中小企业、专业市场为一体的板块经济,发挥产业集群效应,提升区域板块经济的产业水平。进一步放宽投资领域,完善社会化服务体系,鼓励、支持和引导民营经济加快发展。

　　强化经济运行调度,跟踪分析企业生产经营情况,在技术改造、产品开发、设备进口和财政贴息等方面加大支持力度,积极帮助解决资金、原材料供应和市场开拓等方面的困难,促进工业经济快速健康发展。

　　(三)加大“三农”工作力度,在社会主义新农村建设上实现良好开端

　　在严格保护耕地、稳定粮食产量、提高粮食品质的基础上,引导农民积极发展果蔬、花卉、中药材等高产、优质、高效、生态、安全农业,促进优势农产品生产基地建设。发展优质小麦700万亩,优质抗虫棉380万亩,西瓜、大蒜、芦笋、食用菌、蚕桑、中药材、花卉、杞条等特色农产品生产基地500万亩。大力发展“两牛两羊”,积极推广林牧结合和规模化饲养,加快推进林业、畜牧产业化进程。以银香伟业有机牛奶开发为重点,在发展有机循环农业上实现新突破。全年规模饲养畜禽达到4000万只(头),新增造林面积30万亩。加快农业标准化工作,加强农业技术推广、检测检验和动植物病虫害防控体系建设,进一步提高农产品质量安全水平。以建设有机循环农业示范区为目标,尽快启动黄河故道区综合开发工程。

　　综合运用财政、信贷、农业综合开发等政策,重点扶持一批带动能力强的农业产业化龙头企业,全年新增规模以上龙头企业28家。大力发展以农副产品加工业为重点的农村二、三产业,提高农村工业化和农业产业化水平。培育农村合作经济组织,推广龙头企业、合作组织与农户有机结合的组织形式,让农民从产业化经营中得到更多的实惠。

　　继续实施农村劳动力转移培训阳光工程,进一步强化农民职业技能培训。加强对劳务输出的指导和服务,实行劳务派遣制度,努力扩大劳务输出规模,打造特色劳务品牌,提高劳务输出的层次和水平。力争全年培训农民和转移农村劳动力分别达到20万人以上。

　　加快“空心村”改造,搞好乡村环境综合整治,改善村容村貌。继续大力实施“村村通”工程,巩固提高“双通”成果,实现所有行政村通油路、通客车;完善黄河以桥代渡工程,基本完成现有危桥改造任务;抓好重点水利工程和农田水利配套建设,增强抗旱除涝能力;改造现有农村危房9万平方米;加大农村改厕、改水力度,积极推广沼气,改善农村生产生活环境。

　　加大公共财政支农力度,让更多的公共服务深入农村、惠及农民。认真落实“两免一补”政策,加快农村中小学校危房改造。在巩固东明县省级新型农村合作医疗试点的基础上,将牡丹区、单县、定陶纳入省级新型农村合作医疗试点,全面铺开农村医疗救助工作,完善农村卫生监督和防保体系,改善农村医疗卫生状况。加强农村文化场所建设,提高农村有线电视覆盖率,新增通有线电视行政村1000个。在完善“两证一卡”制度、保障农村特困群体基本生活的基础上,逐步扩大低保范围,提高保障水平,力争农村社会养老保险参保人数净增2万人以上,五保户集中供养率达到60%以上。

　　(四)以商贸流通为重点,加快服务业发展步伐

　　抓紧编制服务业特别是商贸物流业发展规划,引导商业网点合理布局。进一步提高恒盛市场、康庄乐园市场和牡丹商业步行街的运营水平,加快鲁西南建材市场、施乐超市、天苑商都、香格里拉购物广场、康庄乐园二期工程建设,启动东方红商业步行街改造工程,推进农资、建材、服装、鞋城、水果等专业市场的整合提高,培育壮大曹县桐木制品、郓城杨木制品、鄄城中药材和人发等特色专业市场。专业批发市场交易额力争达到265亿元以上。

　　大力发展特许经营、代理制、电子商务等新型流通业态,促进传统服务业升级换代。依托现有仓储设施、运输条件和大型工业企业,积极发展第三方物流。努力扩大菏泽口岸的吞吐能力。积极鼓励大型超市向农村、社区延伸,实行连锁经营,健全农村商品流通体系和城市社区商业便民服务体系。力争新型流通业态经营额突破30亿元。进一步提升现有宾馆、酒店的服务档次和质量,开发建设名吃城和小吃一条街,以此聚集人气、拉动消费,繁荣发展晚间经济。

　　加快发展新兴服务业。深化农村信用社改革,积极筹建地方商业银行,争取外地金融机构来菏落户,增强金融机构对经济发展的保障能力。积极开发牡丹观赏、红色旅游、文物古迹、水浒文化、黄河生态等旅游资源,加大宣传促销力度。围绕老城区开发和城中村改造,加快推进安居工程和东部大型居住区建设。进一步搞好策划运作,努力提高牡丹花会、林交会、房交会的办会水平,着力打造特色会展品牌。加快发展医疗、保健、园艺、保安、养老和家政等社区服务业,建立健全满足居民需求的新型社区服务体系。重视发展技术咨询、代理、信息、律师、会计、审计、设计和资产评估等现代服务业。

　　(五)积极推进城市和基础设施建设,改善经济社会发展条件

　　依据城市总体规划,抓紧搞好分区规划和专业规划,增加规划深度,提高详规覆盖率。结合城市综合开发,注重搞好城市设计,努力提升建筑物的品位和档次。同时,搞好县城总体规划和详细规划,加快编制建制镇特别是中心镇规划,形成配套的城市规划体系。

　　加大城市建设力度。推进城市道路、管网、绿化、供水、供气、供热、安居、污水处理等工程建设。高标准配套改造西安路,建设北环路,打通部分断头路,完善“两环、四横、六纵”的交通框架。加快环城公园、赵王河公园和牡丹园配套改造工程建设,开工建设中华大剧院等大型公益项目。完成1000套经济适用住房建设任务。提升市区“绿、亮、清”工程标准,完善污水管网,实现清污分流;加强公厕、垃圾中转站和垃圾处理厂配套建设;大力度开发街头绿地,大规模增加城市绿量,大幅度提高城市绿化水平,着力打造以“花城、水邑、林海”为特色的平原森林城市风貌。全面完成8县污水处理厂及配套管网建设,确保市区、县城污水集中处理率分别达到60%和30%。提升县城和中心镇规划建设水平,完善城镇功能,增强吸纳农村人口、带动农村发展的能力。

　　依法规范城市经营。对所有建设用地依法实行有偿出让,努力增加政府经营收益,确保全年实现政府经营纯收益8亿元,其中市区5亿元。全面推进城市各类有形资产和无形资产经营,通过市场化运作和城市资源的优化配置,吸引社会资本和外资参与城市基础设施、公益事业和服务项目建设,努力形成政府投资引导、国家开发银行低息贷款支持、社会投资广泛参与的多渠道城市建设投资格局。

　　进一步提高城市管理水平。以创建国家级卫生城市为目标,巩固提高市区环卫体制改革成果。强化城市规划执法,严禁零星插建、乱占乱建行为。充分发挥城市管理综合执法机构的职能作用,大力推进城乡环境综合整治。规范建筑市场,严格工程招标,强化质量监督,建设精品工程,防止发生新的工程款拖欠现象。继续开展“做文明市民、树行业新风”活动,增强群众的城市意识,完善城市管理长效机制。

　　大力推进以交通、水利为重点的基础设施建设。加快建设菏泽至兰考、济南至菏泽高速公路(菏泽段)工程,开工建设德州至商丘、东明至新乡高速公路(菏泽段)工程;加快巨野至东明等国省干线公路的升级改造,积极打造国家级公路交通主枢纽城市。开工建设洙水河航道一期工程,推进平原水库、骨干河道治理和黄河标准化堤防二期工程建设,做好地方铁路和菏泽机场的前期工作。

　　(六)继续深化各项改革,大力提高对外开放水平

　　以转变政府职能为重点推进行政管理体制改革。加快政企分开、政资分开、政事分开、政府与市场中介组织分开,强化社会管理和公共服务职能。分类推进事业单位改革。

　　继续深化国有企业特别是市属企业改革,推动国有资本有序退出一般性竞争领域。加快国有企业对外重组,增强国有经济的综合竞争能力。进一步完善企业法人治理结构,建立健全现代企业制度。加强国有资产监管,确保国有资产保值增值。

　　在坚持依法治税,强化税费征管的同时,不断调整优化支出结构,强化财政监管。集中财力加快公共财政建设。继续深化部门预算管理,全面实行国库集中收付改革,完善政府采购制度。建立健全债务防范机制,实行乡镇举债审核报批制度,严禁新增政府债务。严格规范政府投资行为。

　　进一步巩固农村税费改革成果,大力推进农村综合改革。严格执行国家免征农业税和各项支农惠农政策,加快推进乡镇机构、农村义务教育体制、县乡财政体制等项改革,特别是以实行编制实名制为基础,强化编制和财政的双控机制。

　　瞄准国外大财团、大企业,策划、论证一批好项目,有针对性地搞好境外招商引资,提高利用外资水平。调整外贸扶持政策,优化进出口产品结构,在巩固果蔬、食品、裘革制品、木制工艺品等传统产品出口的同时,扩大医药、化工、机电产品出口,提高出口产品的质量和档次。大力发展加工贸易,提高加工深度,延长增值链条。扩大外派劳务规模,鼓励有条件的企业到境外投资办厂。

　　(七)大力发展科技教育事业,加强人才队伍建设

　　进一步完善“以县为主”的农村义务教育管理体制,努力增加教育投入,改善办学条件,确保编内教师工资按国家标准发放。加强师资队伍特别是校长队伍建设,激发学校活力,提高教育教学质量。力争全市小学、初中适龄儿童入学率分别达到99%和98%。努力扩大高中阶段教育规模。把发展职业教育作为推进工业化、城市化和建设新农村的最佳结合点,整合现有中等职业教育资源,引导更多的社会资本投向职业教育,争取组建1-2所高等职业技术学院,每县建设改造一所高水平的职业技术教育中心,力争办成几个具有较大规模和影响的职教示范基地。继续加强菏泽学院后续工程和重点学科建设,支持菏泽医专新校区建设。

　　强化企业在科技进步和自主创新中的主体地位,引导企业加强与高等院校、科研院所的产学研联合,引进或联合开发新产品、新技术、新工艺。鼓励支持有条件的大型企业和优势企业建立自己的技术研发机构,配强技术研发力量,跟踪最新技术动态,组织开展技术攻关。积极组织开展群众性技术创新活动。力争省、市级企业技术中心分别新增2家和5家以上。加强对知识产权的保护,加大企业研发投入的税前抵扣,为企业自主创新提供政策保障。

　　紧紧抓住培养、引进和用好人才三个环节,加强人才队伍建设。在积极培养实用人才的同时,引导企事业单位主要面向西部和东北地区,着力引进一批我市经济社会发展急需的高层次专业技术人才,特别是学科带头人、中青年学术骨干和企业经营人才。全面落实各项人才政策,深化人事制度改革,不断完善业绩评价考核体系,形成有利于造就人才、吸引人才、用好人才、留住人才的良好社会环境。

　　(八)注重资源节约和环境保护,加快生态市建设

　　按照减量化、再利用、资源化的原则,大力推进节能节水节地节材,加强资源综合利用。着力降低电力、化工、造纸、建筑等高耗能行业和企业的资源消耗水平。严格执行国家土地政策,着力推进土地节约和集约利用。积极推广农业节水灌溉技术,开展创建节水型企业、节水型社区活动。提高全社会节约意识,营造节约为荣的良好社会风尚。从今年开始,市里将定期公布各县区单位生产总值、单位工业增加值能耗及污染物排放情况。

　　深入实施“碧水蓝天”工程,继续开展“整治违法排污、保障群众健康”专项活动,全面加强工业点源和城区大气污染治理,大力推进城市统一供热、供气工程,严厉查处各类环境违法行为。严格执行环境影响评价和“三同时”制度,从源头上控制新的环境污染和生态破坏。加强对小流域、沙化区的综合治理,实行测土施肥和秸秆还田,推广农膜替代产品,加快生态农业建设。以黄河滩区、黄河故道、四旁植树和农田林网建设为重点,深入开展全民义务植树活动,鼓励支持社会力量参与造林开发。继续实施“绿色通道”工程,进一步完善环城林带,巩固提高市区绿化水平。力争到年底全市林木覆盖率达到29.5%,市区空气质量良好率达到88%。

　　(九)加快发展社会事业,努力开创和谐社会建设新局面

　　做好就业和社会保障工作。认真落实下岗失业人员再就业扶持政策,多渠道开发就业岗位,千方百计扩大就业和再就业,力争年内新增城镇就业8.4万人,城镇登记失业率控制在4%以内。综合运用行政、经济和法律等措施,加大社会保险费征缴力度,扩大养老、医疗、失业、工伤等基本社会保险覆盖面,适当提高城镇低保标准。建立农村居民最低生活保障制度和失地农民基本生活保障制度,保障困难群众的基本生活。完善社会救助体系,发展慈善事业,建立健全困难群众医疗、教育、住房等专项救助制度。重视保障妇女儿童权益,积极发展残疾人和老年人事业。

　　加强民主法制建设。进一步完善基层民主管理制度,大力推行政务公开、村务公开、厂务公开。认真办好人大代表议案、建议和政协委员提案。支持审判机关和检察机关依法独立行使审判权和检察权。深入开展普法宣传教育,做好法律服务和法律援助工作。发挥工会、共青团、妇联等人民团体的桥梁纽带作用。认真做好民族、宗教和侨务工作。积极做好双拥和兵役工作,支持部队建设。

　　推进平安菏泽建设。高度重视人民来信来访工作,健全矛盾排查调处机制,及时处理群众反映的突出问题。不断完善专门机关、专业力量与群众相结合的治安防控体系,坚持开展严打整治斗争,加强社会治安综合治理,确保社会政治和治安秩序持续稳定。坚持安全第一、预防为主、综合治理,全面落实安全生产责任制,认真开展专项治理活动,坚决防止重特大安全事故发生。进一步整顿和规范市场经济秩序,加强食品药品市场监管,创造公平公正、诚实守信、健康有序的市场环境,确保人民群众生命和财产安全。

　　全面发展各项社会事业。积极推进文化大市建设,深化文化体制改革,加快发展文化事业和文化产业,繁荣新闻出版、广播影视、文化艺术,创造更多更好适应人民群众需求的优秀文化产品。切实加强文化市场管理,营造扶持健康文化、抵制腐朽文化的社会环境。完善公共卫生和医疗服务体系,加强疾病预防控制,特别是重大传染病防治工作,提高疾病预防控制和医疗救治服务能力;加强药品市场监管和医德医风建设,认真研究并逐步解决群众看病难、看病贵问题。坚持计划生育的基本国策,创新计划生育工作机制,稳定人口低生育水平,提高出生人口素质,有效治理出生人口性别比偏高的问题。广泛开展全民健身运动,提高竞技体育水平。继续做好档案、史志、人防、气象、防震减灾工作,促进各项社会事业全面发展。

　　各位代表!人民群众是我们的“衣食父母”,为人民群众搞好公共服务是各级政府的神圣职责。我们一定要牢固树立执政为民的理念,怀着深厚的感情为群众做好事、办实事、解难事。尽管我们不可能在短时期内把所有问题都解决好,但必须从今年起把工作精力尽可能地向这方面集中,把财力物力最大限度地向这方面倾斜,从解决群众最急需解决的问题入手,从能够办好的事情做起,抓住不放,持之以恒,争取每年都办成几件与群众利益最直接的实事,让广大人民群众得到更多实惠,共享改革发展成果!

　　四、政府自身建设的主要措施

　　做好今年的工作,为“十一五”发展开好局、起好步,确保实现“突破菏泽”的战略目标,是历史赋予全市各级政府光荣而艰巨的崇高职责。各级政府要适应新形势新任务的要求,切实加强自身建设,努力提高推动经济社会发展的能力和水平。

　　要加快转变政府职能。进一步转变经济调节和市场监管方式,切实把政府经济管理职能转到主要为市场主体服务和创造良好发展环境上来。特别要更加注重履行社会管理和公共服务职能,更加重视城乡公共设施建设和解决民生问题,更加重视公共安全建设,提高政府应对公共危机的能力。继续推进政府工作提速,健全完善审批服务中心的运行机制,切实减少办事程序,提高服务效率和服务质量。

　　要全面推进依法行政。认真贯彻依法治国基本方略,全面实施依法行政纲要,把依法行政贯穿于政府工作的全过程,严格依法行使权力、履行职责,依法管理经济社会事务,善于运用法律法规调整各种社会关系和利益关系,善于在法制轨道上解决各种矛盾和问题,全面提高依法行政水平。把科学决策、民主决策作为政府决策的基本准则,建立和完善重大事项集体决策制度、专家咨询和评估制度、决策听证和公示制度、决策责任追究制度,提高决策水平,努力使各项决策更加切合实际,更加符合群众利益。大力推行和规范政务公开,发挥好市长公开电话、政府网站、政策法规咨询中心的服务功能,保障人民群众的知情权、参与权、选择权和监督权。进一步改善行政执法,强化行政执法监督,健全执法人员责任追究制度,做到严格执法、公正执法、文明执法、高效执法。自觉接受人大及其常委会的监督,政协和各民主党派、人民团体的监督,广大人民群众和舆论监督。

　　要切实加强廉政建设。坚定不移地抓好党风廉政建设和反腐败工作,建立健全教育、制度、监督并重的惩治和预防腐败体系,严格落实党风廉政建设责任制,加强领导干部廉洁自律。重点加大在土地批租、工程招投标、政府采购、产权交易等领域的反腐倡廉力度。充分发挥监察、审计等监督机关的作用,加强对行政机关运用权力、履行职责的监督检查。坚持纠建并举、综合治理,坚决纠正学校乱收费、截留挪用教育经费以及医疗服务、城镇房屋拆迁、农民负担等方面的不正之风,继续加大对“三乱”的治理力度,切实解决以权谋私、与民争利和损害群众利益的问题,优化发展环境。

　　要努力改进工作作风。牢固树立科学发展观和正确的政绩观,把发展的热情、科学的态度和务实的作风统一起来,大力弘扬求真务实精神,大兴求真务实之风,力戒心浮气躁,防止急于求成,不提脱离实际的高指标,不喊哗众取宠的空口号,不搞劳民伤财的虚假数字,不建沽名钓誉的“形象工程”,坚持讲实话、鼓实劲、干实事、求实效,各项工作都要经得起实践的检验、群众的检验和历史的检验。大力精简会议和文件,把更多的时间和精力用在调查研究和工作落实上。进一步完善决策目标、执行责任、考核监督“三个体系”,对重要工作、重点项目建立严格的目标责任制,实行全程跟踪、即时监控,确保各项具体工作措施的落实。以贯彻实施《公务员法》为契机,对各级各部门公务员进行严格教育、严格管理、严格监督,努力建设一支政治坚定、业务精通、清正廉洁、作风优良的公务员队伍。各位代表!菏泽的发展正处在一个重要的历史机遇期。全市人民对今后的发展寄予了厚望。我们一定要在省委、省政府和市委的正确领导下,高举邓小平理论和“三个代表”重要思想伟大旗帜,全面贯彻落实科学发展观,紧紧依靠和团结全市人民,万众一心,埋头苦干,开拓创新,为建设繁荣、富裕、和谐、文明的社会主义新菏泽而努力奋斗!
